# Liubao tea as a functional fermented food: multi-omics insights into its modulation of lymphatic endothelial cell metabolism

**DOI:** 10.3389/fnut.2026.1759992

**Published:** 2026-05-18

**Authors:** Xiaoxiao Huang, Zhibiao Tan, Chuan Lu, Jianwen Xu

**Affiliations:** 1 Department of Rehabilitation Medicine, The First Affiliated Hospital of Guangxi Medical University, Nanning, Guangxi, China; 2 The First Clinical Medical School, Guangxi Medical University, Nanning, Guangxi, China; 3 Department of Rehabilitation Medicine, Guigang City People's Hospital, Guigang, Guangxi, China

**Keywords:** metabolism-inflammation, Liubao tea, lymphatic endothelial cells, multi-omics, secondary lymphedema

## Abstract

**Background:**

The “metabolism-inflammation” vicious cycle is an important driving factor for lymphatic dysfunction in secondary lymphedema (SL). Among them, lymph endothelial cells (LECs) act as a key regulatory hub. Liubao tea (LBT), a traditional post-fermented black tea, has anti-inflammatory, anti-fibrotic and lipid-lowering effects. However, whether the effects of LBT can extend to the lymphatic system, especially whether it functions by regulating the metabolism and immune function of LECs, still needs further clarification.

**Methods:**

This study combined single-cell transcriptomics, bulk transcriptomics, network pharmacology, and molecular docking to systematically identify potential genes related to LBT in LECs. Subsequently, the effects of the LBT extract on the functions of human lymphatic endothelial cells (HLECs) and the expression of target genes were verified using the Cell Counting Kit-8 (CCK-8), wound healing experiments, and real-time quantitative polymerase chain reaction (RT-qPCR).

**Result:**

The single-cell analysis revealed that the proportion of endothelial cells (ECs) in SL was significantly reduced (from 1.09% to 0.54%), but the expression of protein kinase Cβ (PRKCB) in the ECs showed a significant increase [fold change (FC) = 24.30, log_2_FC = 4.60, *p* = 1.44 × 10^−7^], and the positive rate increased from <1% to 12%. Multigene integration analysis revealed that PRKCB was the only gene that overlapped among all the differentially expressed genes (DEGs) in large-scale transcriptomes, the DEGs specific to ECs, and the predicted targets of LBT. Protein–protein interaction (PPI) network analysis revealed that PRKCB interacts with the nuclear factor κB kinase inhibitor β subunit (IKBKB), the phosphatidylinositol-4,5-bisphosphate 3-kinase catalytic subunit γ (PIK3CG), and glycogen synthase kinase 3β (GSK3B). Molecular docking results indicated that multiple LBT components (especially tirilose) have a strong affinity for PRKCB (minimum binding energy: −9.3 kcal/mole). *In vitro* experiments demonstrated that the extract of LBT (50 μg/ml) significantly reversed the decline in the survival rate and migration ability of HLECs caused by lipopolysaccharide (LPS; *p* < 0.01 and *p* < 0.001), and downregulated the mRNA expression of PRKCB (*p* < 0.05), and upregulated the mRNA expression of PROX1 (*p* < 0.05).

**Conclusion:**

This study reveals that LBT may ameliorate LEC dysfunction in lymphedema by targeting PRKCB to disrupt the bidirectional “inflammation-metabolism” vicious cycle. These findings provide a scientific basis for the use of LBT as a functional fermented food in lymphedema intervention and open new avenues for targeting PRKCB with natural products in the treatment of inflammation-related lymphatic diseases.

## 1 Introduction

The lymphatic system is a key participant in the body's circulation and immune activities (1). Its main functions include maintaining tissue fluid balance and preventing abnormal fluid accumulation; clearing cellular debris and metabolic waste from tissues; assisting in the transport of immune cells; and promoting the absorption of lipids in the intestines (2). These functions work together to maintain the stability of the internal environment and support normal immune regulation. Once lymphatic drainage is impaired, it often indicates a dysfunction in lymphatic function. Abnormalities in the lymphatic system can lead to various pathological changes such as lymphedema, metabolic disorders, and cardiovascular and cerebrovascular diseases (3).

Secondary lymphedema (SL) is a chronic and progressive disease characterized by impaired lymphatic drainage, leading to interstitial fluid accumulation, adipose tissue deposition, and fibrosis (4, 5). Its global prevalence and incidence vary by region, and currently approximately 250 million people are affected (6). SL is commonly associated with recurrent infections, trauma, surgery, obesity, and damage or blockage of normal lymphatic vessels caused by malignant tumors and their related treatments (such as surgery, radiotherapy, chemotherapy, etc.) (7). Because the lymphatic system is unable to clear inflammatory mediators and immune cells from the lesions, persistent inflammation and tissue fibrosis occur (8). SL not only affects the patient's appearance but can also cause complications such as pain, limited mobility, and infection, severely impacting the patient's quality of life and mental health (9).

Despite its significant clinical importance, current treatment options for lymphatic dysfunction primarily rely on physical compression and surgical intervention, which are mostly palliative and cannot restore the biological function of the lymphatic vessels. Currently, complete decongestive therapy (CDT), including compression bandaging, manual lymphatic drainage (MLD), and exercise, remains the standard conservative treatment (10, 11). However, the long-term efficacy of CDT is limited by poor patient compliance, particularly during the maintenance phase, where adherence to self-management practices is often suboptimal (12, 13). Therefore, developing novel intervention strategies that can restore lymphatic function at the biological level has significant clinical value.

Metabolic homeostasis is fundamental to normal physiological function and its dysregulation is primarily characterized by abnormalities in glucose and lipid metabolism, accompanied by chronic low-grade inflammation (14, 15). Riboflavin (vitamin B2), as a precursor of flavin adenine dinucleotide (FAD) and flavin mononucleotide (FMN) , activates key pathways in lipid, protein, and carbohydrate metabolism, and is essential for maintaining energy balance homeostasis (16). Disruption of this homeostasis, manifested as oxidative stress, lipid accumulation, and insulin resistance, activates pro-inflammatory signaling cascades such as nuclear factor kappa-B (NF-κB), leading to the production of inflammatory cytokines (17, 18). Conversely, persistent low-grade inflammation impairs insulin signaling, reduces mitochondrial oxidative capacity, and promotes ectopic lipid deposition, thereby exacerbating metabolic dysregulation (19, 20). Notably, riboflavin accumulation has been shown to support energy homeostasis and meet mitochondrial demands under conditions of cellular stress (21), and high-dose riboflavin treatment has been demonstrated to remodel energy metabolism, improve mitochondrial ultrastructure, and reduce metabolic dysfunction in preclinical models (22). This bidirectional “meta-inflammatory” crosstalk forms a self-reinforcing vicious cycle that drives the pathogenesis of various diseases, including lymphatic dysfunction (5, 23).

In recent years, the focus of research on SL has shifted from traditional mechanisms of inflammation and fibrosis to the field of lipid metabolism. This shift can be supported by the large number of published literature during the period from 2022 to 2025 (5, 24–26). It should be noted that the lymphatic system itself is the main pathway for lipid transport. The intestinal lymphatics are responsible for transporting most of the dietary long-chain fatty acids, making the intestinal lymphatic axis a key hub where nutritional perception and systemic metabolic integration converge (27).

Against this backdrop, endothelial cells (ECs) are regarded as crucial regulatory hubs for metabolic and immune homeostasis. Among them, lymphatic endothelial cells (LECs), as the core subpopulation responsible for lymphatic return, play a key role in lipid transport, immune cell migration, and inflammatory signal transduction (28, 29). Prospero-related homeobox 1 (PROX1) is a master transcription factor that determines and maintains LEC fate and represents one of the most specific markers of LECs. Its expression level directly reflects the lineage identity and functional status of LECs (30). As the core cells responsible for lymphatic drainage, LECs are subjected to the synergistic effects of metabolic stress and inflammatory activation under pathological conditions, leading to dysfunction and further exacerbation of lymphatic impairment (31). Studies have shown that the metabolic state of LECs directly influences lipid deposition, immune homeostasis, and the structural integrity of lymphatic vessels (32). Dietary strategies such as reducing saturated fats have been shown to improve lymphatic function (33, 34). Additionally, a comprehensive lifestyle intervention that includes a Mediterranean diet and exercise can improve lymphedema outcomes (35).

Functional fermented foods, as important factors in regulating metabolism and inflammation, have received considerable attention in recent years (36, 37). Liubao tea (LBT) is a traditional post-fermentation black tea originating from the southern part of China's Lingnan region (mainly Guangxi), with a production history of over 1,500 years (38). Unlike unfermented tea, LBT undergoes a unique post-fermentation process driven by microbial communities (such as Eurotium cristatum), resulting in unique bioactive components such as theabrownins, polyphenols, and microbial metabolites (39, 40). High-throughput sequencing analysis revealed that Eurotium and Aspergillus are the dominant fungal genera during the fermentation process, with Eurotium cristatum being the representative species (39, 41). Previous studies have shown that polyphenolic compounds in LBT, including flavonoids such as catechins and quercetin, as well as phenolic acids such as ellagic acid, can inhibit the NF-κB and mitogen-activated protein kinase (MAPK) inflammatory signaling pathways and reduce the expression of pro-inflammatory cytokines such as tumor necrosis factor-alpha (TNF-α) and interleukin-6 (IL-6) (42). Meanwhile, these components can activate the AMP-activated protein kinase (AMPK) pathway, regulate phosphatidylinositol 3-kinase (PI3K)/protein kinase B (AKT) and peroxisome proliferator-activated receptor gamma (PPARγ) signaling, promote fatty acid oxidation, inhibit lipid synthesis, and improve cellular energy metabolism (43).

However, existing research has mainly focused on classical metabolic organs such as the liver and adipose tissue (38). Whether the effects of LBT can be extended to the lymphatic system, and in particular, whether it acts by directly regulating the metabolic and immune functions of ECs, remains to be further elucidated.

This study employed an integrated strategy combining differential expression analysis, network pharmacology, and single-cell transcriptomics to screen for potential key regulatory targets in ECs, and identified protein kinase C beta (PRKCB) as a potential key target through which LBT regulates ECs function. *In vitro* experiments further validated the regulatory effects of LBT on PRKCB expression and endothelial cell function.

Therefore, this study aims to explore the potential role of LBT in improving endothelial dysfunction and its regulation of PRKCB expression, providing a theoretical basis for the application of LBT as a functional dietary component in the prevention and treatment of SL.

## 2 Materials and methods

### 2.1 Study design

**Figure 1:**
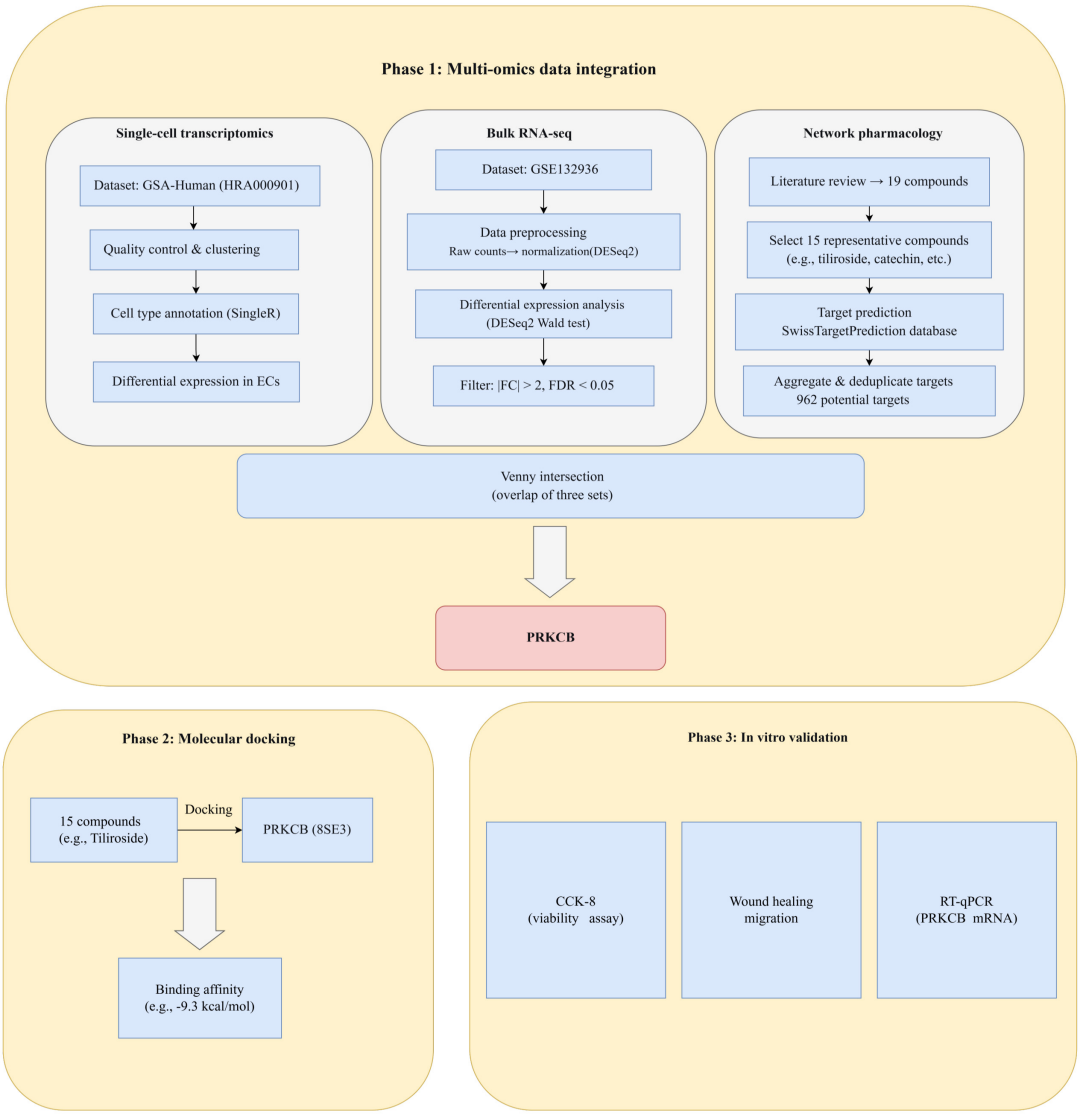
Experimental workflow of the study.

The overall study design is summarized in Figure 1. This study consisted of three main phases: (1) multi-omics data integration (single-cell and bulk transcriptomics, network pharmacology); (2) molecular docking and target validation; (3) *in vitro* experiments (CCK-8, wound healing, RT-qPCR).

### 2.2 Single-cell RNA sequencing (scRNA-seq) data processing and analysis

#### 2.2.1 scRNA-seq data source and preprocessing

The scRNA-Seq data used in this study were obtained from the publicly released dataset by Liu et al. (44). This dataset includes adipose tissue samples from five patients with SL and four healthy controls. The raw sequencing data (in FASTQ format) can be accessed from the National Genomics Data Center of China (CNCB) Human Genome Sequence Archive (GSA-Human) under the accession number HRA000901 (https://ngdc.cncb.ac.cn/gsa-human/browse/HRA000901). The original matrix is stored in RDS format and contains 81,947 cells and 26,414 genes.

#### 2.2.2 Quality control of scRNA-seq data

Firstly, we constructed a single-cell object using the “Seurat” (45) package (version 5.4.0). The initial filtering criteria were: each gene was expressed in at least 10 cells (min.cells = 10), and each cell detected at least 200 genes (min.features = 200). Then, the mitochondrial gene proportion (percent.mt) was calculated, and the low-quality cells were filtered based on the number of genes (nFeature_RNA, ranging from 200 to 6,000). The threshold for the mitochondrial gene proportion was set at < 20%. Based on the quality control, we also addressed the issue of environmental RNA contamination by using the decontX algorithm (from the celda package, version 1.24.0) to perform decontamination processing separately for each sample (46). For each sample, after calculating the adaptive threshold based on the distribution of contamination proportions (using the interquartile range method, ranging from 0.10 to 0.30), we removed the highly contaminated cells and retained the decontaminated expression matrix for subsequent analysis. Subsequently, we also used “DoubletFinde” r (version 2.0.6) to conduct double-cell (doublets) detection for each sample (47). The optimal pK value was determined through parameter scanning, and corrections were made by combining the estimated double-cell rate and the proportion of identical double cells. Double cells were identified and removed (for samples with a small number of cells, “scDblFinder” was used as an alternative method). After completing the quality control, we standardized the data (NormalizeData, using LogNormalize method, with scaling factor = 10,000), performed high-variable gene screening (FindVariableFeatures, using vst method, with nfeatures = 1,500), and scaled the data (ScaleData). We also conducted dimensionality reduction through Principal Component Analysis (PCA, with npcs = 20). To reduce batch effects, we used the Harmony algorithm (version 1.2.0) to perform integration correction based on the sample source (sample_id). Finally, based on the dimensionality reduction results, we constructed a cell adjacency graph (FindNeighbors) and conducted cluster analysis (FindClusters, resolution = 0.2), and then used the UMAP method for visualization. All downstream analyses were performed based on the selected principal component dimensions (PCs).

#### 2.2.3 Cell type annotation and differential expression analysis

To perform cell type annotation, this study utilized the “SingleR” tool (version 2.10.0) for automatic annotation (48). Using the HumanPrimaryCellAtlasData (celldex package, version 1.18.0) as the reference database, the SingleR function was employed to assign cell type labels to each cell population. The annotation results are added to the metadata of the Seurat object, which will be used for subsequent analysis. To investigate the transcriptional differences between the disease group and the control group in various cell types, we conducted a differential expression analysis based on the annotated cell types. First, the identity of each cell was defined as the combined label of “sample group _ cell type”. Subsequently, for each cell type, the cells from the disease group and the control group were separately extracted, and the “FindMarkers” function in the Seurat package was used to conduct the differential gene screening (45). During the analysis, the threshold for differential expression was set to an absolute average log_2_ fold change (avg_log_2_FC) greater than 0.1, and a Bonferroni-adjusted *p*-value (*p*_val_adj) of less than 0.05 was used as the criterion for statistical significance. For cell types satisfying the criteria of having at least three cells per group and exhibiting significantly differentially expressed genes (DEGs), the resulting list of differentially expressed genes is saved in text format to a specified output directory for subsequent analysis.

### 2.3 GEO dataset acquisition and differential expression analysis

This study downloaded the dataset GSE132936 from the Gene Expression Omnibus (GEO) database (49). This dataset contains adipose-derived mesenchymal stem cells (ASCs) from 10 patients with malignant tumor-associated LE of the limbs. Adipose tissue from the LE site and normal adipose tissue from the upper abdomen of the same patient were used as controls. The raw sequencing data were generated using the GPL20795 platform (Illumina HiSeq X Ten; Illumina, Inc., San Diego, CA, USA).

It should be noted that the transcriptome data used in this study originated from adipose-derived mesenchymal stem cells in the fat tissue of lymphedema sites, reflecting the molecular changes in the microenvironment of lymphedema tissues rather than the specific mechanisms of the tumor tissues themselves. Previous studies have shown that SL caused by different etiologies (such as tumor-related, obesity-related, post-infection, etc.) exhibits high consistency in key pathological processes, mainly including chronic inflammation, dysfunction of LECs, remodeling of adipose tissue, and fibrosis, etc. (24, 25). Therefore, this dataset provides an appropriate analytical framework for exploring the generally significant molecular characteristics in lymphedema. Moreover, the core conclusion of this study was independently verified through *in vitro* experiments (an inflammation model induced by LPS), and it was not dependent on the specific etiology of this dataset. For data preprocessing and differential expression analysis, the raw count expression matrix was normalized using the online platform Microbioinformatics (https://www.bioinformatics.com.cn). Differential expression analysis was subsequently performed using the DESeq2 algorithm (50). Differentially expressed genes (DEGs) were identified based on the following criteria: |fold change (FC)| > 2, corresponding to more than a two-fold change in expression, and a false discovery rate (FDR) < 0.05. Furthermore, to prevent numerical overflow, the lower limit of the extremely small *p*-value is truncated to 1e−100. Volcano plots were generated to visualize DEGs, with significance thresholds set at |log_2_FC| > 1 and false discovery rate (FDR) < 0.05. For heatmap visualization, the top 30 DEGs with the largest absolute |log_2_FC| were selected. The expression data were normalized using the *Z*-score method.

### 2.4 Acquisition of target points for the active components of LBT

To identify the potential active components and their targets of LBT, we conducted a systematic literature search, which identified 19 representative compounds (51)^.^ Detailed information on these compounds (including names, chemical classes, molecular formulas, and reported biological functions) is provided in Supplementary Table 1. Considering the coverage of the target prediction database and the predictability of the compounds, 15 compounds with high content and representative features were selected for subsequent target prediction. The 15 compounds selected for target prediction were caffeine, catechin gallate, chrysin, ellagic acid, epicatechin-3-O-gallate (ECG), eriocitrin, hemiphloin, kaempferol, luteolin, naringenin, quercetin, rutin, theophylline, tiliroside, and trifolin. The common names of these compounds were entered into the PubChem database (https://pubchem.ncbi.nlm.nih.gov) to retrieve their canonical SMILES strings (52). Based on the SMILES structures, potential targets were predicted using the SwissTargetPrediction database (http://www.swisstargetprediction.ch) with the species restricted to *Homo sapiens* (53). The predicted targets for all 15 compounds were aggregated and duplicates were removed, resulting in 962 potential targets of LBT active components. The target information (including compound names and target gene names) is organized in Supplementary Table 2 and served as the basis for subsequent network pharmacology analysis.

### 2.5 Multisource data integration analysis and candidate target screening

The three gene sets (overall transcriptomic DEGs, EC-specific DEGs, and predicted targets of LBT active components) were imported into the Venny 2.1 online tool (https://bioinfogp.cnb.csic.es/tools/venny/index.html) to obtain their intersection. These intersecting genes were defined as candidate key targets through which LBT may regulate EC function to ameliorate SL. This gene list will be used for subsequent protein–protein interaction (PPI) network analysis and *in vitro* experimental validation.

### 2.6 Construction of protein–protein interaction networks

To systematically analyze the interactions among the target protein set, we constructed a protein-protein interaction network using the GeneMANIA online platform (https://genemania.org/) (54). This platform integrates a variety of functional association data including physical interactions, co-expression, genetic interactions, co-localization and pathway sharing. In terms of specific operation, the aforementioned potential candidate genes obtained through multi-omics integration screening are input into this database. GeneMANIA, based on its built-in, well-validated association network, automatically generates a functional association network for these genes and their potential interacting proteins. The final obtained network diagram provides a clear and intuitive illustration of the potential regulatory relationships among these candidate targets and their positions within the functional modules.

### 2.7 Molecular docking

Molecular docking verification was performed by downloading the molecular structures of the main active ingredients from the Pubchem database (https://pubchem.ncbi.nlm.nih.gov/) and saving them in SDF format. Download the 3D crystal structure of the target protein from the PDB database (http://www.rcsb.org/) and save it as a PDB file. Use PyMOL software to perform dehydration and hydrogenation operations on the protein and ligand, save the results as a PDB file, and use the Getbox Plugin to obtain the docking pocket parameters. The processed protein and active ingredient files were imported into AutoDock Tools 1.5.6 and converted to PDBQT format. Molecular docking was then performed using AutoDock Vina 1.1.2 (55). Finally, the molecular docking results were visualized using PyMOL 2.6.0 software (56).

### 2.8 Preparation of LBT extract

Approximately 100 g of LBT were weighed, added to 1 L of deionized water, and heated to boiling in a 100 °C water bath for 45 min. After extraction, 250 ml of the tea infusion was taken, frozen into a solid, and then freeze-dried using a freeze dryer (Model: FreeZone 2.5L, Labconco, USA). Finally, approximately 7 g of freeze-dried tea extract powder was obtained. Freeze-drying has been shown to effectively preserve the bioactive components of tea and plant extracts and is suitable for *in vitro* cell experiments (57–59). Then, 100 mg of the lyophilized powder was dissolved in 1 ml of basal culture medium (RPMI-1640, Gibco, USA; Cat. No. 11875093) under sterile conditions to prepare a 1 mg/mL stock solution. The stock solution was sequentially sterilized by filtration through 0.45 μm and 0.22 μm microporous membranes (Millipore, USA; Cat. No. SLHV033RB), aliquoted, and stored at −20 °C in the dark until use. For experiments, the stock solution was diluted with complete culture medium to the desired working concentrations (1–200 μg/ml).

### 2.9 Cell culture

Human lymphatic endothelial cells (HLECs) were purchased from Jinyuan Biotechnology (Shanghai, China; Cat. No. JY336). The cells were cultured in specialized medium for LECs (Zhongqiao Xinzhou, Shanghai, China; Cat. No. ZMY068). Cells were maintained at 37 °C in a humidified incubator with 5% CO_2_ (Thermo Fisher Scientific, USA; Model: Heracell™ 150i). In the experiment, we used cells from the 3rd to 6th generations to ensure a stable cell state. During the cell passage process, 0.25% trypsin-EDTA (Servicebio, Wuhan, China; Cat. No. G4021) and the passage was carried out at a ratio of 1:3.

### 2.10 Cell counting kit-8 (CCK-8) assay

In order to evaluate the cytotoxicity and proliferation effects of the LBT on HLECs, we employed the CCK-8 assay (Meilunbio, Dalian, China; Cat. No. MA0225). Firstly, in the cell toxicity assessment, we seeded HLECs onto 96-well plates (Servicebio, China; Cat. No. CCP-96N) at a density of 5.0 × 10^3^ cells/well and cultured overnight. Cells were then treated with LBT at concentrations of 0, 1, 5, 10, 20, 50, and 100 μg/ml for 24 h. Three technical replicates were performed per concentration, and the experiment was independently repeated three times.

For proliferation assessment, HLECs were seeded at a density of 3.0 × 10^3^ cells/well into 96-well plates and cultured overnight. The following experimental groups were set up: (1) control (normal culture), (2) lipopolysaccharide (LPS) stimulation (1 μg/ml LPS; Solarbio, Beijing, China; Cat. No. L8880), and (3) LPS + LBT intervention (1 μg/mL LPS + 50 μg/mL LBT). Cells were incubated for 0, 24, and 48 h. Three technical replicates were used per group, and the experiment was independently repeated three times.

After treatment, the medium was removed, and the cells were gently washed three times with 1 × PBS (Solarbio, Beijing, China; Cat. No. P1020). Then, 100 μl of fresh serum-free medium containing 10% CCK-8 reagent was added to each well, and the plates were incubated at 37 °C in the dark for 1–4 h. Absorbance was measured at 450 nm using a microplate reader (BioTek, USA). Cell viability (%) was calculated as:


$$\begin{array}{l} \frac{OD_{experimental}-\;OD_{blank}}{OD_{control}-\;OD_{blank}}\times 100\% \end{array}$$

Data analysis and graph generation were performed using GraphPad Prism 9.5.1 software (GraphPad Software, USA).

### 2.11 Wound healing assay

Cell migration was assessed using a scratch wound healing assay. HLECs were seeded into 6-well plates (Servicebio, Wuhan, China; Cat. No. CCP-6H) and cultured until confluence. A uniform scratch was created across the monolayer using a sterile 200 μl pipette tip (Solarbio, Beijing, China; Cat. No. YA0405). After washing twice with 1 × PBS (Solarbio, Beijing, China; Cat. No. P1020) to remove detached cells. The following experimental groups were set up: (1) control (serum-free medium), (2) LPS stimulation (serum-free medium containing 1 μg/ml LPS; Solarbio, Beijing, China; Cat. No. L8880), and (3) LPS + LBT intervention (serum-free medium containing 1 μg/ml LPS + 50 μg/ml LBT). Wound closure images were captured at 0, 24, and 48 h using an inverted microscope (Olympus, Japan). The wound closure rate was quantified using ImageJ software by measuring the scratch width at five different positions per well. The wound closure rate (%) was calculated using the distance method as:


$$\begin{array}{l} \frac{W_{0}-\;W_{t}}{W_{0}}\times 100\% \end{array}$$

*W*_0_ is the initial scratch width at 0 h, and *W*_t_ is the scratch width at the indicated time point (24 h or 48 h).

### 2.12 Real-time quantitative polymerase chain reaction (RT-qPCR)

After 6 h of treatment, total RNA was extracted from HLECs using the EasyPure^®^ RNA Kit (TransGen Biotech, Beijing, China; Cat. No. ER101-01). RNA concentration and purity were assessed using a NanoDrop 2000 spectrophotometer (Thermo Fisher Scientific, USA), with an A260/A280 ratio between 1.8 and 2.0. Subsequently, 1 μg of total RNA was reverse transcribed into complementary DNA (cDNA) using the SweScript All-in-One Blue RT SuperMix for qPCR (with One-Step gDNA Remover; Servicebio, Wuhan, China; Cat. No. G3337). Real-time quantitative PCR was performed using TransStart^®^ Top Green qPCR SuperMix (+Dye I; TransGen Biotech, Beijing, China; Cat. No. AQ132-11) on a Gentier 96E Real-Time PCR System (Tianlong, Xi'an, China). The thermal cycling conditions were as follows: initial denaturation at 95 °C for 2 min, followed by 40 cycles of denaturation at 95 °C for 15 s and annealing/extension at 60 °C for 60 s. A melting curve analysis was performed to verify amplification specificity. β-actin was used as the internal control, and relative gene expression levels were calculated using the 2–ΔΔCt method. All reactions were performed in triplicate (technical replicates) from three independent biological replicates. The primer sequences are listed in Table 1.

**Table 1:** Primer sequences used for qPCR amplification.

Gene	Forward primer (5^′^ → 3^′^)	Reverse primer (5^′^ → 3^′^)	Amplicon (bp)	Tm (°C)
PRKCB	ACGTGGAGTGCACTATGGTG	TCACAAAGTACAGGCGGTCC	120–150	60
ACTB (β-actin)	CCATCGTCCACCGCAAAT	GCTGTCACCTTCACCGTTCC	150	60

### 2.13 Statistical analysis

All statistical analyses were performed using R software (version 4.5.1) and GraphPad Prism 9.5.1 (GraphPad Software, USA). Data are presented as mean ± standard deviation (SD). All tests were two-sided, and *p* < 0.05 was considered statistically significant.

Normality was assessed using the Shapiro–Wilk test. Homogeneity of variance was evaluated using the *F*-test for two-group comparisons and Levene's test for multiple-group comparisons. For comparisons between two groups, Student's *t*-test was applied when data were normally distributed with equal variances; otherwise, the Wilcoxon rank-sum test (Mann–Whitney *U*-test) was used. For comparisons among multiple groups, one-way analysis of variance (ANOVA) was performed for normally distributed data with homogeneity of variance, followed by Tukey's honestly significant difference (HSD) test for *post hoc* multiple comparisons. When data did not meet these assumptions, the Kruskal–Wallis' test was applied.

For multiple testing correction, differential expression analyses were adjusted using the Benjamini–Hochberg method to control the false discovery rate (FDR), with adjusted *q* < 0.05 considered statistically significant. In multiple-group comparisons, Tukey HSD was used for *post hoc* correction following ANOVA, while Bonferroni correction was applied where appropriate in other multiple comparison scenarios.

Effect sizes were calculated to quantify the magnitude of differences. Cohen's *d* was used for *t*-tests (small ≥ 0.2, medium ≥ 0.5, large ≥ 0.8), and *r* = |Z|/√*N* was used for Wilcoxon tests (small ≥ 0.1, medium ≥ 0.3, large ≥ 0.5).

Differential expression analysis of scRNA-seq data was conducted using the Seurat package (FindMarkers function) with a two-sided Wilcoxon rank-sum test. Genes with |log_2_FC| > 0.585 and FDR < 0.05 were considered significantly differentially expressed. Bulk RNA-seq differential expression analysis was performed using DESeq2 with a two-sided Wald test, using thresholds of |FC| > 2 and FDR < 0.05.

For *in vitro* experiments, including CCK-8 assays, wound healing assays, and real-time quantitative polymerase chain reaction (RT-qPCR; relative expression calculated using the 2-^ΔΔCt method), statistical analyses were performed using one-way or two-way analysis of variance (ANOVA) as appropriate, followed by Tukey's or Sidak's multiple comparisons test. Pearson correlation analysis was used to evaluate gene expression relationships (two-sided), and correlation coefficients with 95% confidence intervals (CI) were calculated using the cor.test function. Data are presented as mean ± standard deviation (SD). A *p*-value < 0.05 was considered statistically significant. Statistical significance was denoted as follows: ^*^p < 0.05, ^**^p < 0.01, ^***^p < 0.001, ^****^p < 0.0001.

A detailed methodology checklist is provided in Supplementary Table 3.

## 3 Results

### 3.1 Quality control of scRNA-seq data

This study used single-cell RNA sequencing technology to systematically analyze the cellular composition of the control group and SL group. To assess the quality of the single-cell transcriptome data, we performed statistical analysis on the number of gene features (nFeature_RNA), transcript count (nCount_RNA), and mitochondrial gene proportion (percent.mt) before and after filtering for each sample (Supplementary Figure 1A: before filtering; Supplementary Figure 1B: after filtering). The results showed that the overall distribution of nFeature_RNA and nCount_RNA did not change significantly before and after filtering. The number of genes in most cells remained concentrated between 500 and 6,000, and the sequencing depth was evenly distributed, indicating that the original data already had good complexity and coverage. Regarding the distribution of percent.mt, the proportion of mitochondrial genes in most cells was at a low level (mainly concentrated in the range of 0–10%), suggesting that the overall cell quality was high and no obvious abnormal population with high mitochondrial expression was observed. Although a small number of high percentage.mt cells were present in individual samples, their proportion was low and their impact on the overall analysis was limited. During single-cell quality control, the raw data underwent rigorous screening. Initially, 81,947 cells were obtained, and after quality control, 81,562 high-quality cells were retained, resulting in a cell retention rate of 99.53%. The number of genes remained consistent before and after filtering (26,414), with a gene retention rate of 100% (Supplementary Table 4).

To further improve the accuracy of single-cell data, this study assessed and corrected for environmental RNA contamination. The contamination level analysis results for each sample are shown in Supplementary Table 5. The results showed that the average contamination rate across different samples was generally low to moderate (approximately 0.021–0.137), with most samples falling within the range of 0.02–0.08. The median contamination level was generally low (approximately 0.01–0.03), indicating that the vast majority of cells were minimally affected by contamination. Based on an adaptive threshold (approximately 0.10–0.17), approximately 118–2,674 highly contaminated cells per sample were removed. After removal, each sample retained 2,827–12,728 cells, and contamination correction was successfully completed for all samples. After quality control, we used “DoubletFinder” to identify and remove potential doublets, and the results are shown in Supplementary Table 6. The results showed that the overall proportion of doublets in each sample was within a reasonable range (approximately 1.45%−8.03%), with the doublets rate in most samples concentrated between 5% and 7%, consistent with the expected doublets incidence rate in high-throughput single-cell sequencing. DoubletFinder analysis identified and removed 41–1,022 doublets across all samples. After doublets removal, each sample retained 2,786–11,706 single cells, with zero unassessed cells, indicating that all cells successfully participated in the doublet identification process.

Following a complete quality control process, a total of 67,977 high-quality cells and 26,414 genes were obtained, and 11 major cell clusters were identified (Supplementary Figure 1C). Among them, the control group consisted of 24,420 cells, and the disease group consisted of 43,557 cells.

### 3.2 Single-cell transcriptome maps reveal the changes in ECs in SL

**Figure 2:**
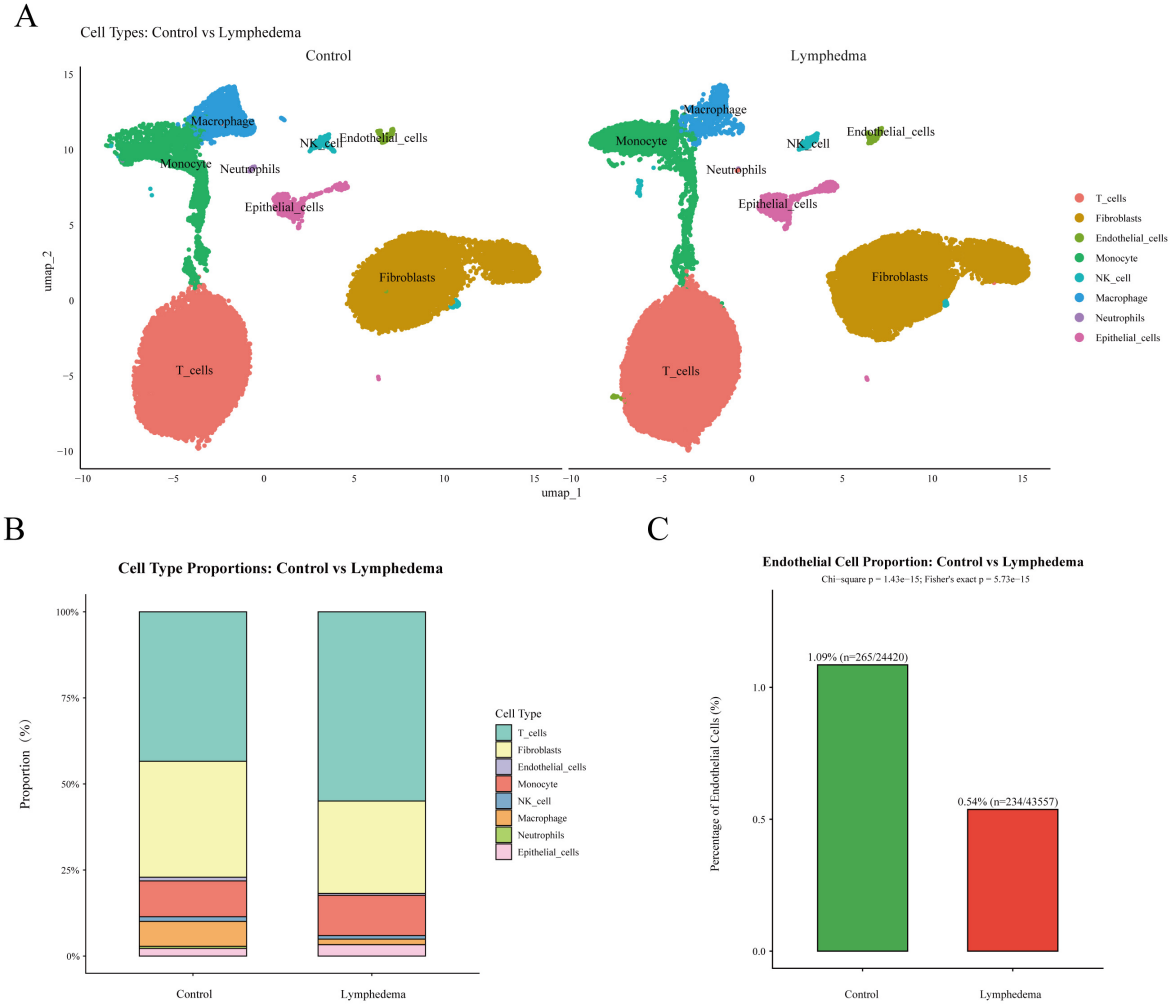
Single-cell transcriptomic atlas reveals altered ECs proportion in lymphedema. **(A)** UMAP visualization of major cell types in control and SL groups, with distinct colors representing different cell subsets. **(B)** Bar plot showing the relative proportions of each cell type in control vs. SL groups, revealing differences in immune and stromal cell composition. **(C)** Comparison of the percentage of ECs between control and SL groups, showing a significant reduction in the SL group. *Note* ECs, endothelial cells; SL, secondary lymphedema; UMAP, uniform manifold approximation and projection.

Through Uniform Manifold Approximation and Projection (UMAP) dimensionality reduction and Louvain clustering, we identified eight major cell clusters, including T cells, fibroblasts, ECs, monocytes, natural killer (NK) cells, macrophages, neutrophils, and epithelial cells (Figure 2A). The cell clusters were clearly separated and showed good comparability between the control and LE groups, indicating high reliability of the cell annotation results.

To further explore cell composition changes associated with SL, we compared the relative proportions of different cell types between the control and SL groups. As shown in Figure 2B, the overall cellular composition exhibited certain differences between the two groups, with immune cells (including T cells, NK cells, monocytes, macrophages, and neutrophils) and stromal cells (including fibroblasts, ECs, and epithelial cells) showing marked trends of change in their proportions between the two groups. Notably, the proportion of ECs was significantly reduced in the SL group (Figure 2C). Quantitative analysis revealed that ECs accounted for 1.09% (265 cells) in the control group, whereas they accounted for only 0.54% (234 cells) in the SL group, with a statistically significant difference (*p* < 0.001). This result suggests that a reduction in the number of ECs or impairment of their function may be an important feature in the development and progression of SL.

### 3.3 Integrated transcriptomic differential expression profiling reveals SL-related transcriptional alterations

**Figure 3:**
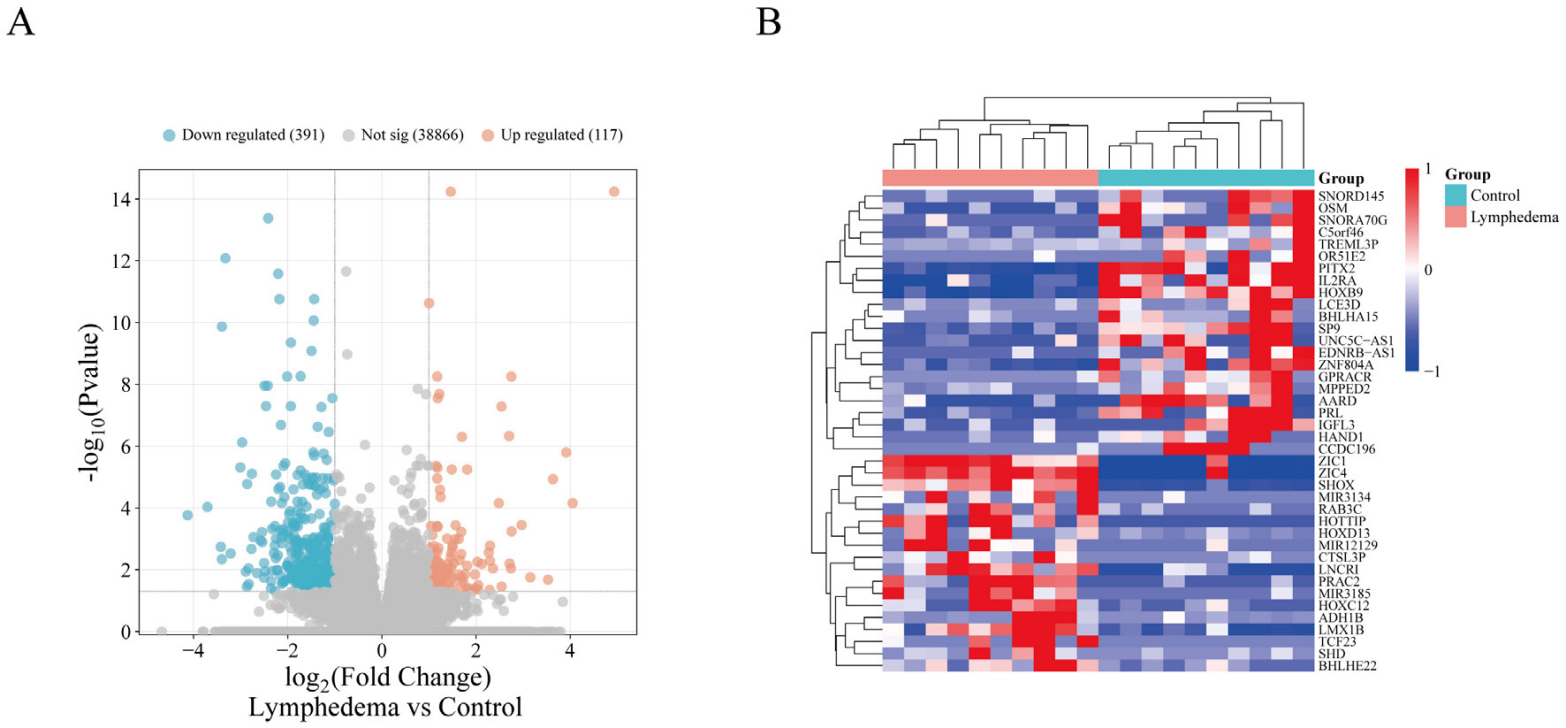
Bulk transcriptomic differential expression analysis. **(A)** Volcano plot of DEGs between SL and control tissues. Up-regulated genes (*n* = 117) are marked in red, down-regulated genes (*n* = 391) in blue, and non-significant ones in gray. The *x*-axis shows log_2_ fold change, and the *y*-axis shows –log10(adjusted *p*-value). **(B)** Heatmap of selected significant DEGs. High expression is coded red, low expression blue. Genes are arranged in rows and samples in columns. Clustering analysis reveals a clear separation between the control and SL groups. *Note* DEGs, differentially expressed genes; FC, fold change; log_2_FC, log_2_ fold change; padj, adjusted *p*-value; SL, secondary lymphedema.

To comprehensively evaluate transcriptional changes in SL at the tissue level, we performed bulk RNA-seq differential expression analysis on the GEO dataset GSE132936 (see Methods 2.2 for details). Using the DESeq2 algorithm with thresholds of |fold change (FC)| > 2 and FDR < 0.05, a total of 1,243 significantly differentially expressed genes (DEGs) were identified, including 586 up-regulated and 657 down-regulated genes. The volcano plot (Figure 3A) clearly illustrates the global distribution of down-regulated (blue) and up-regulated (orange) genes, providing an intuitive basis for systematic comparison between the two groups. The results indicate extensive transcriptomic remodeling between SL tissues and control tissues.

To further reveal the expression patterns of the differential genes, we performed hierarchical clustering analysis on the significant DEGs using a heatmap (Figure 3B). Red indicates increased gene expression, while blue indicates decreased gene expression. Clustering analysis showed that samples from the SL and control groups were clearly separated into two major clusters, demonstrating markedly distinct gene expression profiles between the two groups. These results lay an important foundation for subsequent candidate gene screening and functional mechanism studies. The complete list of DEGs is provided in Supplementary Table 7.

### 3.4 Prediction of targets for bioactive components of LBT

To systematically identify potential targets of LBT, this study selected 15 representative bioactive components based on literature screening, including: Tiliroside, catechin gallate, epicatechin-3-O-gallate, quercetin, luteolin, eriocitrin, hemiphloin, trifolin, kaempferol, chrysin, rutin, ellagic acid, naringenin, theophylline, and caffeine. Target prediction for these compounds was performed using the SwissTargetPrediction database. The predicted targets for each component were integrated and duplicates removed, resulting in a total of 962 potential targets (Supplementary Table 8).

### 3.5 Multi-omics integration analysis identifies PRKCB as a potential target of LBT

**Figure 4:**
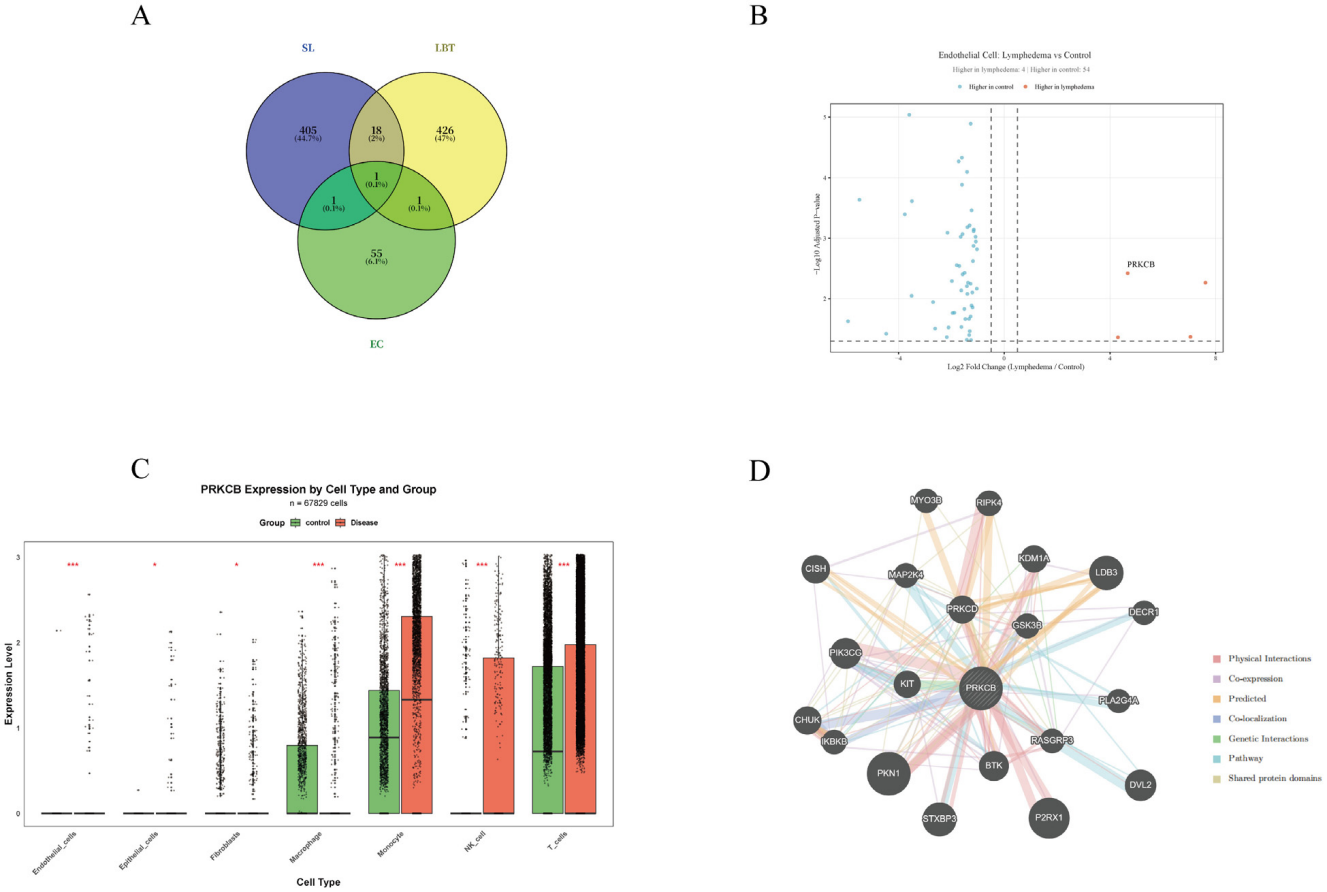
Screening and functional association analysis of PRKCB as a potential target of LBT. **(A)** Venn diagram depicting the overlap among three gene sets: transcriptomic DEGs in SL, EC-specific DEGs, and predicted targets of LBT. **(B)** Volcano plot of EC-specific DEGs, with PRKCB highlighted as a significantly up-regulated gene in the SL group. **(C)** Expression levels of PRKCB in ECs; the SL group shows a marked increase compared to the control group (Wilcoxon rank-sum test, *p < 0.05, **p < 0.01, ***p < 0.001). **(D)** PPI network of PRKCB constructed using GeneMANIA, illustrating extensive connections with multiple signaling-related molecules. *Note* EC, endothelial cell; LBT, Liubao tea; PPI, protein–protein interaction; PRKCB, protein kinase C beta; SL, secondary lymphedema.

To identify key molecules potentially associated with the action of LBT, we performed a multi-source data integration analysis. Three gene sets, including overall transcriptomic differentially expressed genes, EC-specific differentially expressed genes, and predicted targets of LBT active components, were imported into the Venny 2.1 online tool to calculate their intersection. As shown in Figure 4A, PRKCB was the only candidate gene that simultaneously satisfied all three conditions.

### 3.6 Differential expression analysis of ECs reveals significant induction of PRKCB

To investigate the molecular alterations in ECs in SL, we performed differential expression analysis on the EC population. As shown in the volcano plot (Figure 4B), a total of 58 significant DEGs were identified, including 4 up-regulated and 54 down-regulated genes in the SL group (Supplementary Table 9).

Among the four up-regulated genes, PRKCB showed the highest statistical significance (*p*_adj = 0.0038). PRKCB was barely detectable in control ECs (positive rate < 1%), whereas it was markedly induced in lymphedema ECs, with the positive rate increasing to approximately 12% and the expression level elevated by about 24.3-fold (FC = 24.30, log_2_FC = 4.60, *p* = 1.44 × 10^−7^). The other up-regulated genes included CD247 (log_2_FC = 7.61), RUNX3 (log_2_FC = 7.04), and SKAP1 (log_2_FC = 4.30), while down-regulated genes included ABCC9, DIXDC1, DAAM2, among others.

### 3.7 Cell type-specific expression of PRKCB in SL

To further evaluate the expression pattern of PRKCB across different cell types, we compared its expression changes in major cell populations (Figure 4C). The results showed that PRKCB exhibited widespread expression alterations in the SL group, but the degree of up-regulation varied markedly among cell types (Supplementary Table 10). Specifically, PRKCB showed the most prominent up-regulation in ECs the mean expression level in the disease group was increased by approximately 24.3-fold compared with the control group (FC = 24.30, log_2_FC = 4.60, *p*_adj = 1.44 × 10^−7^), and the positive rate increased from nearly zero (0.008%) in controls to 12% in lymphedema. Mild up-regulation was also observed in NK cells (FC = 1.95, log_2_FC = 0.96, *p*_adj = 3.05 × 10^−10^) and monocytes (FC = 1.55, log_2_FC = 0.63, *p*_adj = 4.34 × 10^−46^), whereas T cells showed no significant change (FC = 1.14, log_2_FC = 0.19, *p*_adj = 7.17 × 10^−16^, with a minimal actual difference). In contrast, PRKCB tended to be down-regulated in fibroblasts (FC = 0.36, log_2_FC = −1.48, *p* < 0.05) and macrophages (FC = 0.69, log_2_FC = −0.54, *p*_adj = 3.76 × 10^−24^). These results indicate that the up-regulation of PRKCB in SL is EC-specific, further supporting its role as a potential target of LBT for regulating EC function.

### 3.8 PRKCB PPI network analysis

To further dissect the potential regulatory mechanism of PRKCB in ECs in SL, we constructed a PPI network of PRKCB using the GeneMANIA database (Figure 4D). The results showed that PRKCB occupied a central position in the network and closely interacted with multiple key signaling molecules, including inhibitor of nuclear factor kappa-B kinase subunit beta (IKBKB), phosphatidylinositol-4,5-bisphosphate 3-kinase catalytic subunit gamma (PIK3CG), glycogen synthase kinase 3 beta (GSK3B), Bruton tyrosine kinase (BTK), mitogen-activated protein kinase 4 (MAP2K4), and protein kinase C delta (PRKCD). Notably, the interactions of PRKCB with IKBKB (a key kinase in the nuclear factor kappa-B (NF-κB) pathway), PIK3CG (a core component of the phosphatidylinositol 3-kinase (PI3K)-protein kinase B (AKT) pathway), and GSK3B (a regulator of glucose metabolism) suggest that PRKCB may act as a hub connecting inflammatory signaling and metabolic regulation, thereby participating in the modulation of EC function.

### 3.9 Molecular docking predicts the binding ability of LBT components to PRKCB

To evaluate the potential interaction between bioactive components of LBT and PRKCB, molecular docking was performed to predict the binding affinity of the selected major chemical constituents to PRKCB (PDB ID: 8SE3). Binding energy is an important indicator for assessing the stability of ligand-receptor binding; generally, a lower (more negative) binding energy indicates a stronger affinity between the ligand and the target protein. The molecular docking results showed that several bioactive components of LBT exhibited strong binding affinities to PRKCB (Table 2). Among them, Tiliroside showed the highest affinity, with a binding energy of −9.3 kcal/mol; Catechin gallate and Epicatechin-3-O-gallate also displayed strong binding activity, with binding energies of −8.7 kcal/mol and −8.1 kcal/mol, respectively. Additionally, flavonoids such as Quercetin (−7.9 kcal/mol), Luteolin (−7.8 kcal/mol), and Eriocitrin (−7.7 kcal/mol) also demonstrated good binding capabilities (binding energy ≤ −7.5 kcal/mol). These results indicate that PRKCB is a potential direct target of multiple bioactive components of LBT, with Tiliroside and its related catechin derivatives exhibiting the best intervention potential.

**Table 2:** Molecular docking binding affinities of Liubao tea compounds with PRKCB.

Compound	Binding affinity (kcal/mol)
Tiliroside	−9.3
Catechin-gallate	−8.7
Epicatechin-3-O-gallate	−8.1
Quercetin	−7.9
Luteolin	−7.8
Eriocitrin	−7.7
Hemiphloin	−7.6
Trifolin	−7.5
Kaempferol	−7.4
Chrysin	−7.3
Rutin	−7.3
Ellagic-acid	−7.2
Naringenin	−7.1
Theophylline	−5.2

**Figure 5:**
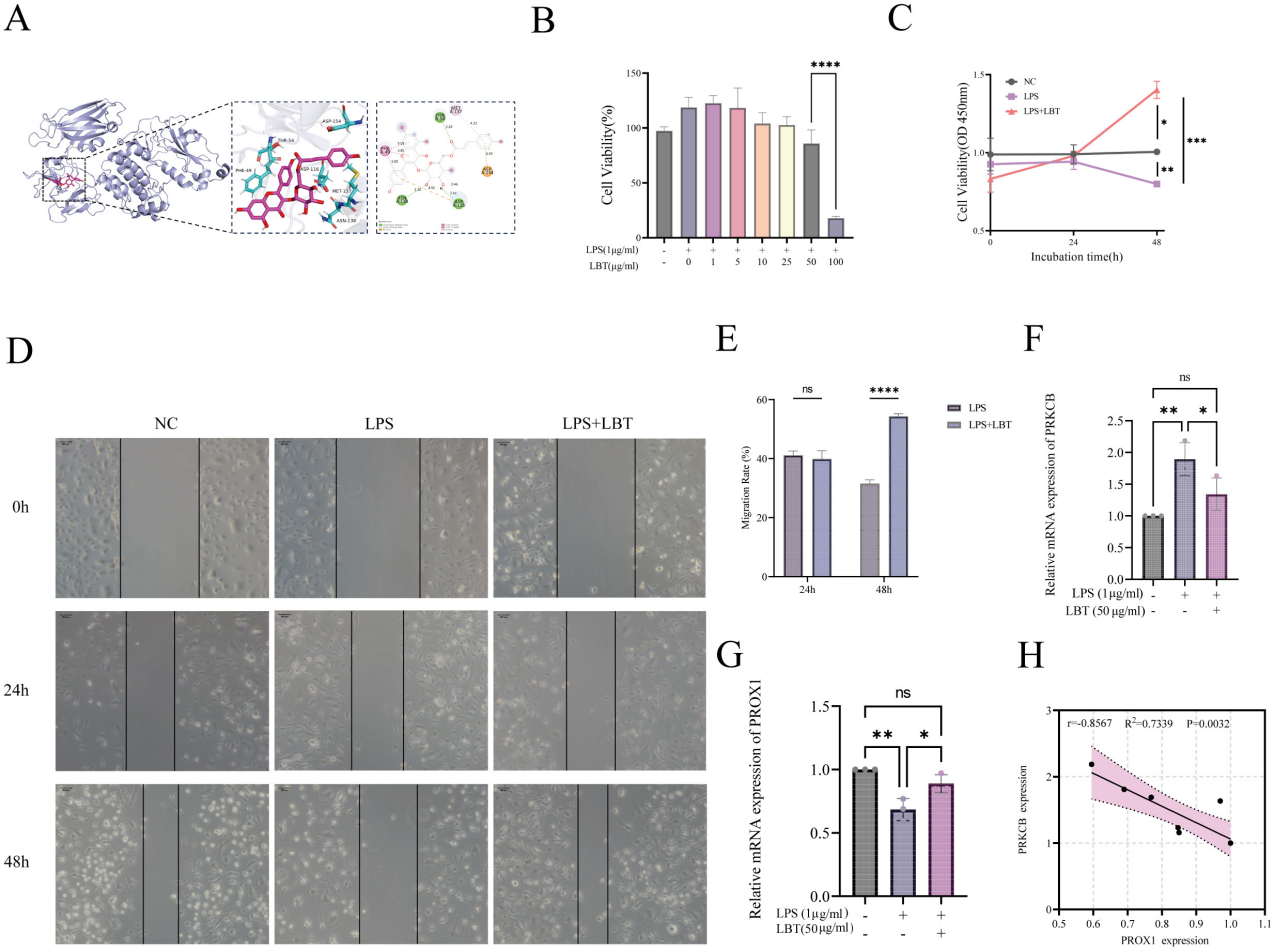
LBT ameliorates endothelial dysfunction under inflammatory conditions. **(A)** Molecular docking analysis showing the binding mode of tiliroside within the active pocket of PRKCB (PDB ID: 8SE3). **(B)** Effects of different concentrations of LBT on HLECs viability after 24 h incubation. **(C)** Effects of LBT on HLECs viability under LPS stimulation at different time points (0, 24, and 48 h). **(D)** Representative images of wound healing assay in HLECs treated with NC, LPS, and LPS+LBT at 0, 24, and 48 h. Scale bars = 200 μm. **(E)** Quantitative analysis of cell migration rate at 24 h and 48 h. Data are presented as mean ± *SD* (*n* = 3). Statistical analysis was performed using two-way ANOVA followed by Sidak's multiple comparisons test. LBT significantly increased migration compared with the LPS group at 48 h (****p < 0.0001), while no significant difference was observed at 24 h (ns). **(F)** Relative mRNA expression of PRKCB in HLECs under different treatment conditions. **(G)** Relative mRNA expression of PROX1 in HLECs under different treatment conditions. **(H)** Correlation between PRKCB and PROX1mRNA expression in HLECs. Data are presented as mean ± *SD* (*n* = 3). Statistical analyses were performed using one-way ANOVA (for **B** and **F**) or two-way ANOVA (for **C** and **E**) as appropriate, followed by Tukey's or Sidak's multiple comparisons test. ns, not significant; *p < 0.05; **p < 0.01; ***p < 0.001; ****p < 0.0001. *Note* EC, endothelial cell; LBT, Liubao tea; PPI, protein-protein interaction; PRKCB, protein kinase C beta; SL, secondary lymphedema; NC, negative control (untreated); LPS, lipopolysaccharide; LPS+LBT, LPS plus LBT extract.

As shown in Figure 5A, the molecular docking visualization of the binding mode of Tiliroside with PRKCB (PDB ID: 8SE3) revealed that the interaction mainly relies on key residues such as ASP-154, THR-54, PHE-49, ASP-116, MET-137, and ASN-138, which may stabilize the ligand binding through hydrogen bonds, hydrophobic interactions, or π-π stacking. Moreover, the ligand also forms extensive van der Waals contacts with numerous surrounding residues, suggesting that the binding interface involves multiple domains. This result demonstrates that Tiliroside can form specific interactions with the active site of PRKCB, providing a structural basis for targeting PRKCB by bioactive components of LBT.

### 3.10 The effect of LBT extract on HLECs viability

To evaluate the effect of LBT extract on the viability of HLECs, the CCK-8 assay was performed under different treatment conditions (Figures 5B, C). The results showed that when treated with LBT alone, low to moderate concentrations (1–50 μg/ml) did not significantly inhibit cell viability; instead, a slight promoting trend was observed at some doses, indicating good biocompatibility of LBT within this concentration range. However, when the concentration increased to 100 μg/ml, cell viability was significantly decreased ( ^****^p < 0.0001), suggesting that a high dose of LBT exerts certain cytotoxicity (Figure 5B). Accordingly, 50 μg/ml was selected as the intervention concentration for subsequent experiments.

In the LPS-induced inflammatory model, HLEC viability showed a time-dependent decline. Compared with the LPS group, cell viability in the LPS+LBT group was significantly increased at 48 h (^*^p < 0.05, ^***^p < 0.001), indicating that LBT could partially reverse LPS-induced cell damage. Moreover, no significant differences were observed among groups at 24 h, whereas differences gradually emerged at 48 h, suggesting a time-dependent protective effect of LBT (Figure 5C).

### 3.11 Effect of LBT extract on HLECs migration ability

To evaluate the effect of LBT extract on the migration ability of HLECs, a wound healing assay was performed to observe migration at different time points (Figures 5D, E). The results showed that the migration ability of the LPS-treated group was significantly lower than that of the control group, with a marked inhibitory effect observed especially at 48 h (Figure 5E). Quantitative analysis revealed that, compared with the LPS group, the migration rate of the LPS + LBT group was significantly increased at 48 h (^****^p < 0.0001). No significant difference was observed between the LPS and LPS + LBT groups at 24 h (Figure 5D).

### 3.12 LBT reverses LPS-induced PRKCB upregulation and PROX1 downregulation in HLECs

To assess whether treatment had an overall effect on PROX1 and PRKCB expression, we performed one-way ANOVA. The analysis showed significant differences among groups for both genes [PROX1: *F*_(2, 6)_ = 18.25, *p* = 0.0028, *R*^2^ = 0.8588; PRKCB: *F*_(2, 6)_ = 13.87, *p* = 0.0056, *R*^2^ = 0.8222]. We then used Tukey's *post-hoc* test to compare individual groups. Compared with the NC group, LPS treatment markedly reduced PROX1 expression (mean difference = 0.3154, *p* = 0.0024) and increased PRKCB expression (mean difference = −0.8952, *p* = 0.0048). Adding LBT together with LPS significantly reversed these changes: PROX1 was restored (mean difference = −0.2043, *p* = 0.0196) and PRKCB was lowered (mean difference = 0.5536, *p* = 0.0412) relative to the LPS-alone group. After LBT co-treatment, both genes reached levels comparable to those in the NC group, with no statistical difference (PROX1: *p* = 0.1706; PRKCB: *p* = 0.1951; Figures 5F, G).

We next examined the relationship between PROX1 and PRKCB expression across all samples. The data passed the Shapiro–Wilk normality test (*p* > 0.05), allowing us to use Pearson correlation. The analysis revealed a strong negative correlation between the two genes (*r* = −0.8567, 95% *CI*: −0.9693 to −0.4467, *R*^2^ = 0.7339, *p* = 0.0032, *n* = 9; Figure 5H).

## 4 Discussion

In this study, by integrating single-cell and bulk transcriptomics with network pharmacology, molecular docking, and *in vitro* experiments, we revealed that LBT may regulate the inflammatory-metabolic signaling network of LECs by targeting PRKCB, thereby ameliorating endothelial dysfunction associated with SL.

The pathogenesis of SL is driven by a vicious circle, wherein impaired lymphatic drainage triggers persistent inflammation and fibrosis, which in turn promotes adipose tissue deposition and exacerbates lymphatic dysfunction (8, 24, 25). Furthermore, accumulating evidence indicates that metabolic dysregulation further amplifies this vicious circle (60). Metabolic abnormalities (including impaired fatty acid oxidation, lipid accumulation, insulin resistance, and disturbed energy homeostasis) interact with and reinforce chronic inflammation, forming a bidirectional “inflammation-metabolism” vicious circle that collectively aggravates LECs dysfunction and tissue damage (33, 61).

In this study, PRKCB emerged as a critical hub specifically linking inflammation and metabolic dysregulation in LECs (62). Our data show that PRKCB is markedly upregulated in ECs from lymphedema tissues (FC = 24.30), with the proportion of PRKCB-positive ECs increasing from < 1% to 12%. Notably, PRKCB interacts with IKBKB (NF-κB activator), PIK3CG (PI3K-Akt component), and GSK3B (metabolic regulator) in ECs, as revealed by PPI network analysis (63–65). This suggests that PRKCB may couple inflammatory stimuli to metabolic reprogramming in ECs, contributing to the “inflammation-metabolism” vicious cycle in SL (62, 66). Importantly, LBT components (especially Tiliroside) bind to PRKCB, and LBT treatment down-regulates PRKCB expression while rescuing EC dysfunction, indicating that targeting PRKCB could disrupt this vicious cycle.

Notably, molecular docking results showed that multiple active components of LBT (such as tiliroside, catechin gallate, quercetin, etc.) can bind to PRKCB, suggesting that LBT may achieve effective regulation of PRKCB through multi-component synergy (67). This multi-target, multi-component characteristic is a natural advantage of fermented tea over single compounds, which may help reduce the side effects of single-target drugs and enhance overall therapeutic efficacy (68). Furthermore, as a daily beverage, LBT has good safety and compliance, making it a promising adjuvant dietary intervention strategy for the long-term management of SL (38).

To directly verify the effect of LBT on the function of LECs, we used HLECs for CCK-8, scratch wound healing experiments and RT-qPCR detection. LPS is the main component of the cell wall of Gram-negative bacteria and can induce a strong inflammatory response through the TLR4 receptor complex. It is a well-known inflammatory model (69). The results showed that the LBT extract (50 μg/ml) treated alone had no significant toxicity to the HLECs viability, and even showed a slight promoting trend at certain concentrations, indicating its good biocompatibility within this concentration range. In the LPS-induced inflammatory model, LBT treatment could significantly reverse the cell viability decline (*p* < 0.01) and migration impairment (*p* < 0.001) caused by LPS, and this protective effect was time-dependent, being particularly evident at 48 h. Previous studies have shown that LPS activates PKC (including PRKCB) through the TLR4 receptor, subsequently triggering the NF-κB signaling pathway, which is a key mechanism underlying endothelial dysfunction (70, 71). PRKCB not only promotes NF-κB-mediated inflammatory responses but also inhibits PI3K/AKT-dependent eNOS regulation, negatively modulating insulin signaling and glucose metabolism, thereby leading to endothelial metabolic dysfunction (66). In this study, the LBT intervention significantly downregulated the expression of PRKCB mRNA induced by LPS (*p* < 0.05), confirming the negative regulatory effect of the LBT on PRKCB at the transcriptional level. Furthermore, existing studies have shown that the active component quercetin in LBT can reduce the expression of PRKCB (72), which is consistent with our molecular docking results. These *in vitro* functional experiments not only verified the predictions of multi-omics and molecular docking, but also provided direct functional evidence that PRKCB is a key target for LBT in improving endothelial dysfunction in SL.

To further explore the relationship between PRKCB and LEC identity, we examined PROX1 and PRKCB expression by qPCR. As presented in the Results, LPS stimulation markedly decreased PROX1 expression while increasing PRKCB expression, and LBT co-treatment significantly reversed both changes. Pearson correlation analysis across all samples revealed a strong negative correlation between PROX1 and PRKCB expression (*r* = −0.85, *p* < 0.01). This finding is consistent with a previous report showing that PROX1 represses PRKCB transcription through promoter methylation (73), suggesting that the reciprocal expression changes observed in this study may be mechanistically linked.

In summary, this study reveals that LBT may improve LEC dysfunction by targeting PRKCB to disrupt the bidirectional “inflammation-metabolism” vicious cycle in lymphedema. These findings not only provide a scientific basis for the use of LBT as a functional fermented food in lymphedema intervention but also open up new avenues for targeting PRKCB with natural products in the treatment of inflammation-related lymphatic diseases.

## 5 Limitations

This study has several limitations. First, the single-cell data were derived from a limited number of samples (5 SL vs. 4 controls). Although we validated key findings through *in vitro* experiments, future studies with larger sample sizes are needed to enhance statistical power. Second, functional validation of PRKCB was limited to mRNA levels and cellular phenotypes; protein levels and kinase activity were not examined, nor were siRNA or CRISPR knockdown experiments performed to establish causality. Third, the LBT extract used was a crude extract. Although molecular docking suggested that multiple components may target PRKCB, which specific component plays a dominant role *in vivo* remains to be further isolated and identified. In addition, no *in vivo* animal experiments were conducted in this study; the therapeutic efficacy of LBT on SL and the *in vivo* regulatory role of PRKCB still need to be validated in animal models.

Based on the findings of this study, future research can be pursued in the following directions: (1) using PRKCB-specific inhibitors or LEC-specific PRKCB knockout mice to validate the causal role of PRKCB in the pathogenesis of lymphedema; (2) isolating and purifying the active monomeric components of LBT (e.g., Tiliroside) and verifying their direct binding to PRKCB using techniques such as surface plasmon resonance (SPR); (3) establishing a mouse tail lymphedema model to evaluate the *in vivo* efficacy and safety of LBT extract or its monomeric components; and (4) employing phosphoproteomics technology to map the downstream signaling network of PRKCB, thereby further elucidating the molecular mechanisms by which PRKCB regulates the inflammation-metabolism crosstalk in LECs.

## 6 Conclusion

In summary, this study reveals that LBT may ameliorate inflammation and metabolic dysregulation by modulating PRKCB, thereby alleviating LEC dysfunction in SL. PRKCB is significantly upregulated in LECs and interacts with NF-κB, PI3K-Akt, and GSK3B pathways. LBT components bind to PRKCB and down-regulate its expression, rescuing LEC function. These findings suggest that LBT may serve as a functional fermented food for lymphedema management and highlight PRKCB as a promising natural product target. Future studies are needed to confirm causality through genetic and animal models.

## Data Availability

The original contributions presented in the study are included in the article/supplementary material, further inquiries can be directed to the corresponding author.
