# Supplementary material for: Liubao tea as a functional fermented food: multi-omics insights into its modulation of lymphatic endothelial cell metabolism
Source: Front Nutr. 2026 May 18;13:1759992. doi: 10.3389/fnut.2026.1759992 (PMC13222993; doi:10.3389/fnut.2026.1759992)
Supplement: Supplementary file 1 [file Data_Sheet_1.docx]

Supplementary Material


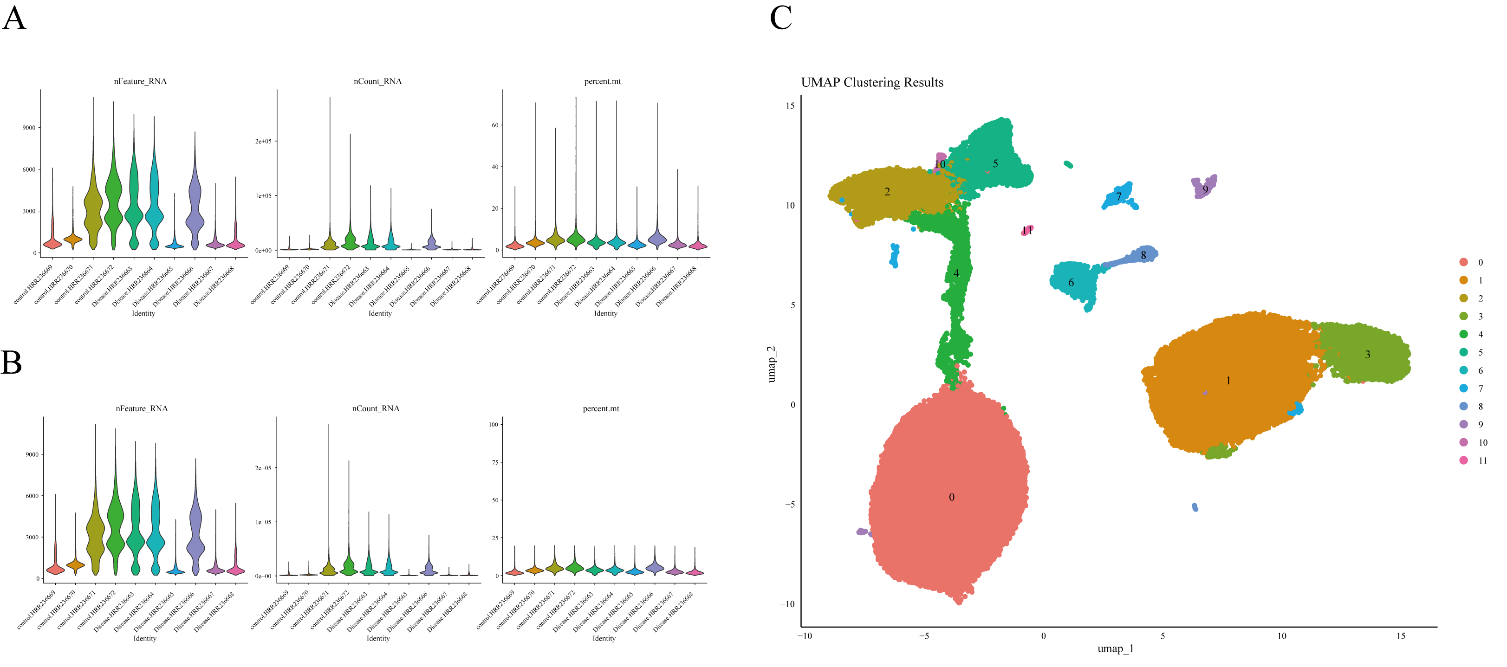


**Supplementary Figure 1. Quality control and cell clustering of single‑cell transcriptomic data.**(A) Distribution of the number of detected genes (nFeature_RNA), transcript counts (nCount_RNA), and the percentage of mitochondrial genes ([percent.mt](https://percent.mt/)) across samples before filtering. (B) Distribution of the same metrics after filtering. (C) UMAP visualization of 67,977 high‑quality cells after quality control, showing 11 major cell clusters (different colors represent different cell types)
